# Frequency, trends, and antecedents of severe maternal depression after three million U.S. births

**DOI:** 10.1371/journal.pone.0192854

**Published:** 2018-02-14

**Authors:** Urbano L. França, Michael L. McManus

**Affiliations:** 1 Boston Children’s Hospital, Department of Anesthesiology, Critical Care and Pain Medicine, Division of Critical Care, Boston, Massachusetts, United States of America; 2 Harvard Medical School, Boston, Massachusetts, United States of America; Harvard Medical School, UNITED STATES

## Abstract

**Background:**

Postpartum depression carries adverse consequences for mothers and children, so widespread screening during primary care visits is recommended. However, the rates, timing, and factors associated with significant depressive episodes are incompletely understood.

**Methods and findings:**

We examined the Healthcare Cost and Utilization Project (HCUP) State Inpatient, Emergency Department, and Ambulatory Surgery and Services Databases from California (2005–2011) and Florida (2005–2012). Within 203 million records, we identified 3,213,111 births and all mothers who had hospital encounters for severe depression within 40 weeks following delivery. We identified 15,806 episodes of postpartum depression after 11,103 deliveries among 10,883 unique women, and calculated an overall rate of 36.7 depression- associated hospital visits per 10,000 deliveries. Upward trends were observed in both states, with combined five-year increases of 34%. First depressive events were most common within 30 days of delivery, but continued for the entire observation period. About half (1,661/3,325) of PPD first episodes occurred within 34 days of delivery, 70% (2,329/3,325) by the end of the second month, and 87% (2,893/3,325) before four-months of the delivery. Women with private insurance were less likely to have hospital encounters for depression than women with public insurance and women with depression were much more likely to have had some kind of hospital encounter at some time during their pregnancies. Rates of depression increased with the number of prenatal hospital encounters in a “dose-dependent” fashion: the rate of depression was 17.2/10,000 for women with no prenatal hospital visits, doubling for women with at least one encounter (34.9/10,000), and increasing 7-fold to 126/10,000 for women with three or more encounters during their pregnancies.

**Conclusions:**

Our findings suggest that (1) hospital encounters for post-partum depression are increasing, (2) screening should begin very early and continue for the first year after delivery, and (3) added attention should be given to women who had hospital encounters during their pregnancies.

## Introduction

Untreated maternal depression can have a lasting impact on the health and well-being of children [1]. For this reason, the U.S. Preventative Services Task Force recommends depression screening for pregnant and postpartum women [2] while the American Academy of Pediatrics includes maternal depression within its recommended well-child care schedule [3]. Supporting this recommendation, the Center for Medicare and Medicaid Services has recently emphasized the importance of maternal depression screening and encouraged both Medicaid and non-Medicaid coverage for the practice [4].

The effectiveness of any screen depends upon its sensitivity, specificity, timing, frequency, and follow-up [5]. In the case of postpartum depression screening, both the U.S. Preventative Services Task Force [2] and Agency for Healthcare Research and Quality [6] acknowledge that important questions remain concerning optimal timing and screening intervals. For pediatricians, timing and frequency are of particular interest, as they seek to efficiently integrate screening into their already-busy practices.

Emergency Department visits and hospitalizations for postpartum depression represent instances where symptom severity reaches a threshold prompting urgent medical attention and where a physician makes a specific diagnosis of depression. Hospital records, therefore, can provide important information concerning incidence, prevalence, antecedents, and timing of severe depression over very large populations. To identify trends in severe depression and inform ongoing screening practices, we used multi-year hospital datasets from two large and geographically distant regions of the United States to investigate the nature of depressive diagnoses that were treated in emergency rooms or admitted to hospitals following more than 3.2 million births.

## Methods

### Study design and dataset

We used the Healthcare Cost and Utilization Project (HCUP), Agency for Healthcare Research and Quality, State Inpatient (HCUP SID), State Emergency Department (HCUP SEDD), and State Ambulatory Surgery and Services Databases (HCUP SASD) from California (2005–2011) and Florida (2005–2012) [7]. HCUP includes information from all inpatient, outpatient, and emergency departments of the hospitals within the state. In total, these datasets contain both demographic and clinical information concerning over 203 million hospital encounters including payer type, medical diagnoses, procedures, condition groupers, urban-rural designation, and other elements. The population of interest was identified using a combination of *International Classification of Diseases*, *Ninth Revision*, *Clinical Modification* (*ICD-9-CM*) codes and *Clinical Classifications Software* (CCS) condition groupers, as described below. California and Florida datasets were selected for the availability of *Revisit Variables* on a large percentage of hospital encounters (>77% in CA and >87% in FL). This permits study of individuals with multiple hospital visits across years and settings using synthetic identifiers. Our work was reviewed and approved by the Boston Children’s Hospital Institutional Review Board.

### Identification of the patients

We first identified the 5,399,936 deliveries in both states using the Medicare Severity-Diagnosis Related Group (MS-DRG) codes for delivery (370–375). We then included only those deliveries for which both Revisit Variables and at least 40 weeks of data were available both pre- and post-delivery. The final dataset contained information concerning 3,213,111 deliveries and 2,554,105 unique women. Using synthetic identifiers, we then collected all ED visits and admissions for each birthing woman during the 40 weeks pre- and post-delivery. The resulting dataset included 2.6 million women and 3,898,444 hospital encounters, defined as either an ED visit or a hospital admission, excluding the deliveries themselves.

We defined “severe” depression as depressive symptoms severe enough to warrant an emergency room visit and/or hospital admission. To identify women with severe depression, we followed the procedure of Savitz, et al., who considered both “definite” and “possible” postpartum depressive conditions [8]. There, “definite” conditions were marked by primary diagnoses with ICD9-CM codes 648.40–648.42, or 648.44 (“Mental disorders of mother complicating pregnancy childbirth or the puerperium”). Recognizing that “puerperium” is timing-based and can be variously applied, they also included as “possible” conditions those with ICD9-CM codes specifying other forms of depression. To similarly capture the full range of pathology, we defined “postpartum depression (PPD)” as any hospital admission or emergency room visit marked by a “definite” code (either as the primary diagnosis or as a secondary diagnosis after a primary mental health diagnosis) and “all depression” as any hospital admission or emergency room visit whose primary diagnosis contained one of the four postpartum depression codes mentioned above or a depression-related code (296.20–296.26, 296.80–296.82, 296.89, 309.0, 309.1, 309.24, 309.28, 309.29, and 311).

Finally, deliveries were grouped based on whether “postpartum depression” or “depression” was included as a primary diagnosis in any ED visit or hospital admission in the 40 weeks after delivery. To determine rates and trends, data were analyzed by state and year. To identify timing and associated conditions, data were aggregated across both states and all years. Visits during the pregnancy preceding each delivery were identified using both synthetic identifiers and mock delivery dates.

### Data analysis

All analyses were performed using Python 3.5, an open-source programming language [9], and the Jupyter interactive environment [10]. Trends were evaluated using linear regression, differences between groups of categorical variables were analyzed using Chi-Squared test, and differences between continuous variables were analyzed using the two-sample Kolmogorov–Smirnov test. Conditional probabilities were calculated directly from the data.

## Results

**Fig 1:**
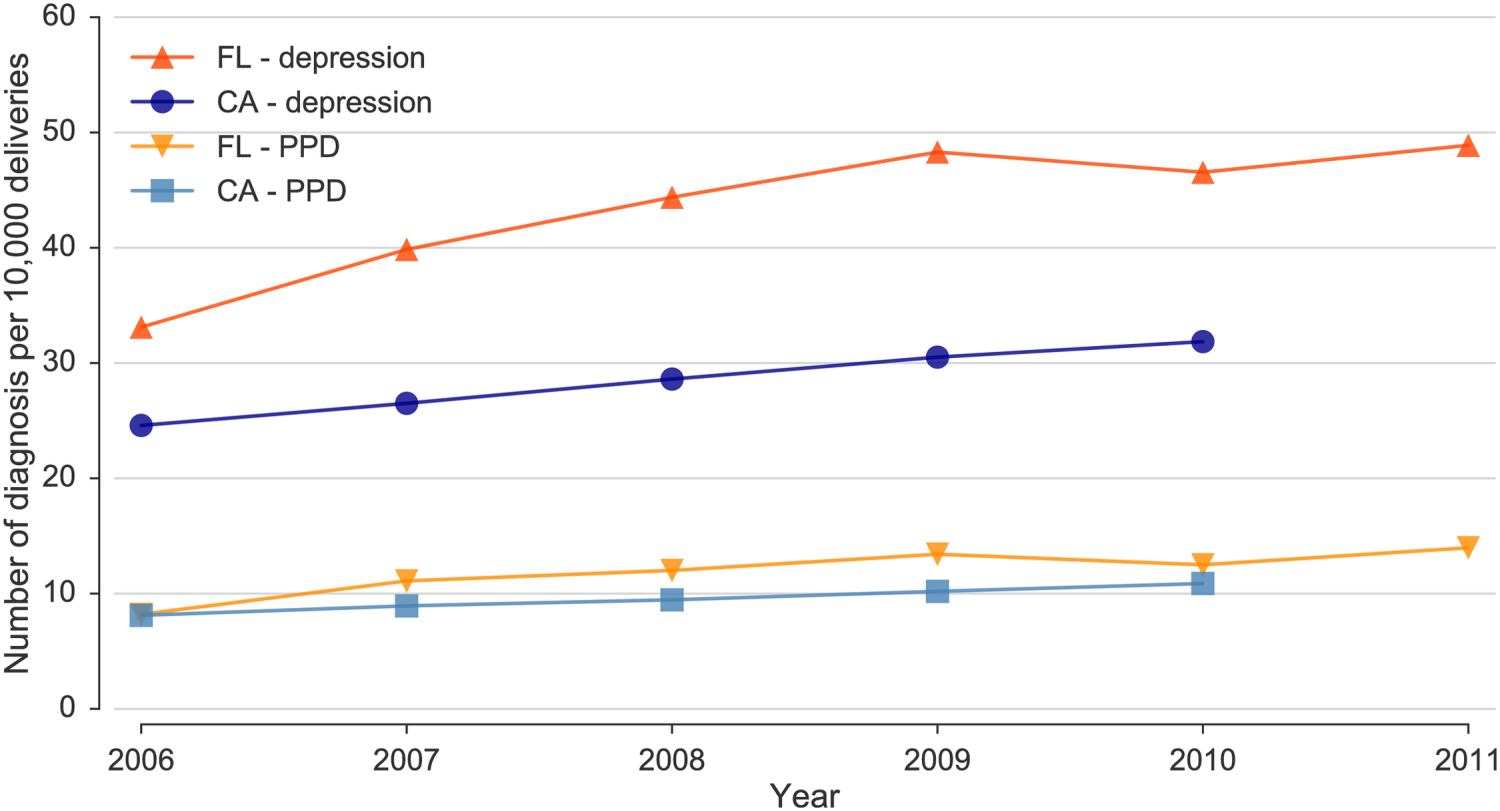
Temporal trends for all depression and PPD diagnoses per 10,000 deliveries for California (p < 0.01 for both trends) and Florida (p < 0.02 for both trends).

We identified 15,806 hospital admissions and emergency visits for depression (8,519 in CA; 7,287 in FL) after 11,103 deliveries among 10,883 unique women. Of these, 3,775 were specifically assigned PPD codes following 3,359 deliveries among 3,325 unique patients. The majority of the encounters, 8,960 (56.7%), consisted of ED visits, 43.2% (6,834) were inpatient admissions, and only 0.01% (12) were in outpatient settings of acute care hospitals. The trends for these conditions are shown in Fig 1, where increases can be seen in both states over the available years. In California during 2006, there were 24.6 hospital encounters per 10,000 deliveries for all depression (PPD: 8.1 per 10,000) and this rate rose 29% to 31.8 per 10,000 deliveries (PPD: 10.9 per 10,000) in 2010 (p < 0.01 for the trends). In Florida, these rates increased 48% from 33.1/10,000 (PPD: 8.2/10,000) in 2006 to 48.9/10,000 (PPD: 14.0/10,000) in 2011 (p < 0.02 for both trends). Combining data from both states, overall 2006–2010 rates rose from 8.1/10,000 to 11.4/10,000 (41%) for PPD and 27.4/10,000 to 36.7/10,000 (34%) for all depression. During the same period, the total number of maternal hospital encounters 40 weeks after delivery increased by 16.9%, from 238,747 (38.4 hospital encounters per 100 deliveries) in 2006 to 258,848 (44.9 hospital encounters per 100 deliveries) in 2010. A higher rate of hospital encounters after delivery, with a higher increase between 2006 and 2010, was observed FL: the rate of hospital encounters was 62.8 per 100 deliveries in FL in 2010, an increase of 19.8% with respect to 2006, compared to 36.1 hospital encounters per 100 deliveries in CA in 2010, an increase of 14.6% during the same period.

Table 1 contains demographic information concerning the general population of women with and without severe depression after delivery. Overall, women with depression were slightly younger and less likely to live in large metropolitan areas than women without depression. Hispanics and Asians were under-represented among deliveries followed by PPD (22.1% and 3.6%) and depression (20.6% and 2.5%) compared to all other deliveries (30% and 7.9%, respectively).

**Table 1:** Characteristics of the women whose deliveries were followed by at least one PPD or depression visit compared to those who were not.

	Deliveries followed by PPD, N = 3,325	Deliveries followed by depression, N = 10,883	All other deliveries, N = 3,202,228
***Age*: *mean ± SD***	27.3 ± 6.6	26.5 ± 6.3	28.1 ± 6.4
***Race***			
White	50.9%	50.3%	42.1%
Hispanic	22.1%	20.6%	30.0%
Black	14.2%	16.9%	12.4%
Asian	3.6%	2.5%	7.9%
Other/Missing	9.1%	9.6%	7.6%
***Insurance***			
Public	51.5%	54.2%	42.6%
Private	37.1%	28.0%	53.4%
Self-pay	5.7%	12.0%	1.9%
Unknown/No charge	5.7%	5.8%	2.1%
***Geography***			
Large metropolitan	67.4%	64.8%	71.0%
Small metropolitan	27.5%	29.5%	25.4%
Micropolitan	2.5%	2.7%	2.3%
Not metropolitan or micropolitan	1.9%	1.9%	1.2%
Missing	0.7%	1.1%	0.1%
***Pregnancy Hospital Encounters***			
No visits	37.8%	32.4%	63.7%
1–2 visits	41.2%	41.5%	30.2%
3–4 visits	11.5%	14.3%	4.3%
5+ visits	9.5%	11.8%	1.8%
***Pregnancy Mental Health Hospital Encounters***			
No visits	87.6%	88.0%	99.4%
1–2 visits	9.5%	9.8%	0.6%
3–4 visits	1.7%	1.3%	< 0.1%
5+ visits	1.2%	0.9%	< 0.1%

### Onset of PPD and depression

**Fig 2:**
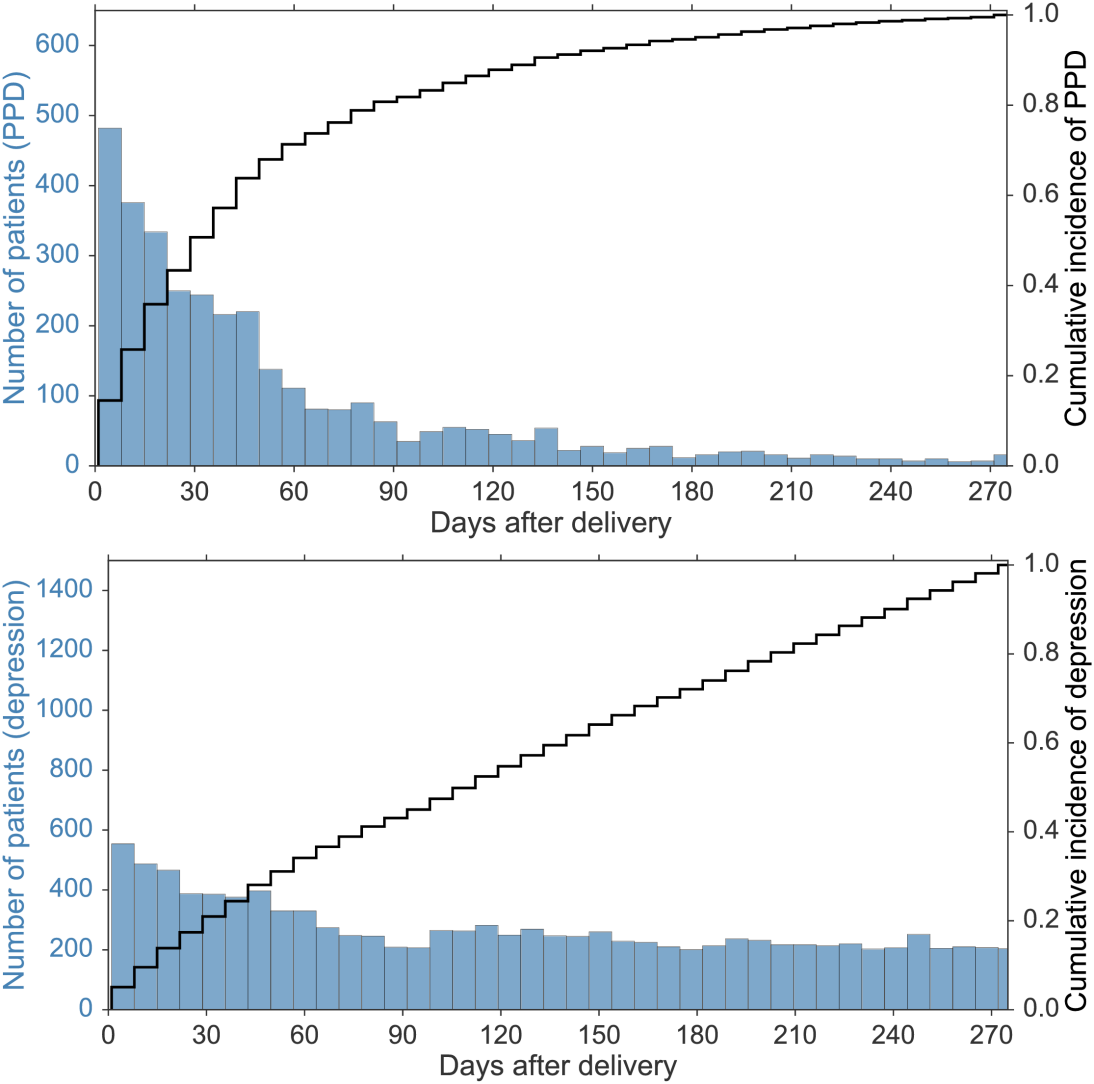
**(*Top panel*)** Timing of onset of the first ED visit or inpatient admission for a specific diagnosis of postpartum depression (PPD). Each vertical bar aggregates a week of hospital encounters. **(*Bottom panel*)** Similar histogram, but for the first hospital encounters for all severe depression.

All data from both states and all years were aggregated to provide information concerning timing of diagnosis and associated conditions. Fig 2 presents the onset of initial diagnoses and the cumulative appearance of severe depression over time. As expected, the top panel of Fig 2 reveals that the PPD diagnosis is most likely to be applied soon after delivery and is highest within the first postpartum week. Considering all depression diagnoses, 5% of first visits were within seven days of delivery (554/10,883), most given a specific PPD diagnosis on days 4–7. There is then a rapid decline in specific PPD diagnoses while, as shown in the bottom panel, initial hospital encounters for all depression continue to occur. About half (1,661/3,325) of PPD first episodes occurred within 34 days of delivery, 70% (2,329/3,325) by the end of the second month, and 87% (2,893/3,325) before four-months of the delivery. However, first visits for all severe depression were more than twice as frequent in the first thirty days compared to the other months after delivery and continued steadily for the remainder of the study period. The nearly diagonal cumulative incidence rate in the bottom panel of Fig 2 suggests that new depression cases can appear anytime within the nine months following delivery.

### Pre-delivery hospital encounters

Women with hospitals encounters for depression after delivery were much more likely to have had some type of hospital encounter during their pregnancies. These prenatal encounters were related to both mental health and non-mental health conditions. The overall pattern of prenatal hospital encounters was essentially reversed for women with and without postpartum depression, with roughly two-thirds of all women having no hospital encounters during their pregnancies but roughly two-thirds of depressed women having had at least one. As detailed in Table 2, the rate of all depression among women who had hospital encounters for any condition prior to delivery was roughly doubled (62.8/10,000).

**Table 2:** Depression rates per 10,000 deliveries and absolute numbers of patients in different data groups based on type of insurance and number of hospital encounters during pregnancy.

Hospital Encounters during pregnancy	Any type of insurance	Private insurance	Public insurance or Uninsured
Any number of visits during pregnancy	33.9 (n = 10,883)	17.8 (n = 3,050)	50.3 (n = 7,198)
***All conditions***			
No visit	17.2 (n = 3,524)	12.1 (n = 1,543)	24.4 (n = 1,779)
1 visit	34.9 (n = 2,157)	22.3 (n = 635)	43.8 (n = 1,399)
2 visits	56.6 (n = 1,463)	38.5 (n = 345)	63.7 (n = 1,038)
3+ visits	126 (n = 3,739)	79.6 (n = 527)	134 (n = 2,982)
1+ visit(s)	62.8 (n = 7,359)	34.2 (n = 1,507)	76.9 (n = 5,419)
***Mental health***			
No visit	29.9 (n = 9,572)	16.7 (n = 2,862)	43.6 (n = 6,177)
1 visit	407 (n = 641)	328 (n = 108)	411 (n = 491)
2 visits	1,147 (n = 312)	1,131 (n = 50)	1,087 (n = 234)
3+ visits	2,232 (n = 358)	1,676 (n = 30)	2,242 (n = 296)
1+ visit(s)	653 (n = 1,311)	481 (n = 188)	663 (n = 1,021)

**Fig 3:**
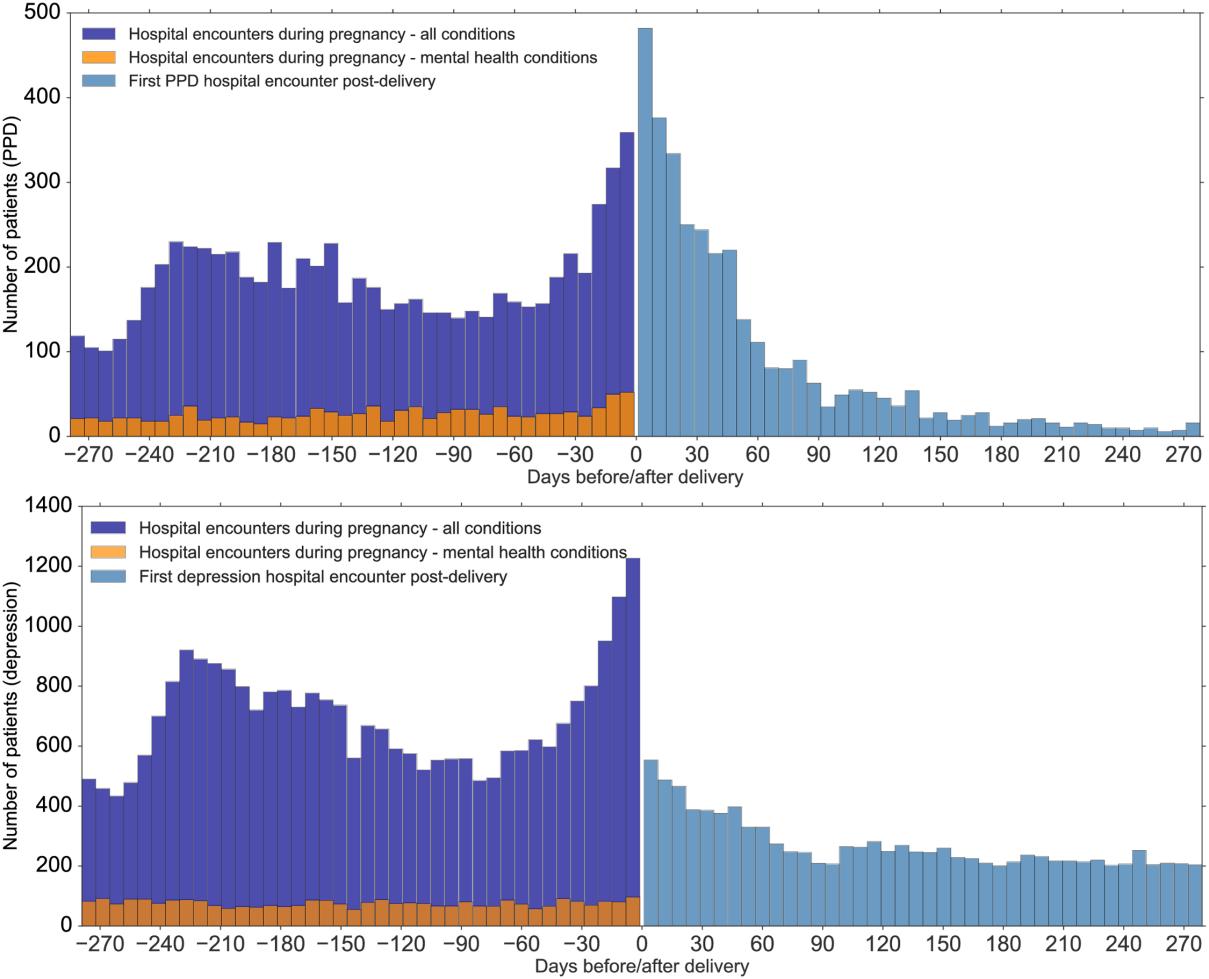
**(*Top panel*)** Histogram of hospital encounters among women who were assigned specific postpartum depression ICD9-CM codes. To the left of zero are visits for all causes prior to delivery and to the right of zero are visits at which the diagnosis of PPD was first assigned. Patients diagnosed with depression in the 9 months after the delivery (light blue bars) are admitted and/or visit the ED department for a diverse set of conditions during pregnancy (dark blue bars), including mental health conditions (dark orange bars). The majority of specific PPD diagnoses are assigned in the first months after delivery, with a long tail of small numbers thereafter. **(*Bottom panel*)**: A similar histogram for all women with depression (see text). Although a specific PPD diagnosis is less common, first visits for depression continue steadily.

Fig 3 aggregates all hospital visits for the weeks before delivery and then PPD (upper panel) or all depression (lower panel) visits for the weeks after delivery. Hospital visits for depression after delivery (light blue) far outnumbered hospital visits for *all* mental health conditions (orange) prior to delivery. Among the women diagnosed with depression after delivery, 8.3% (907) had a hospital encounter for depression during their pregnancies and 12.0% (1,311) had encounters for some mental health condition.

However, the rate of severe depression after delivery also increased substantially with the number of prenatal hospital encounters, regardless of the conditions for which women were seen. Among those with no prenatal hospital encounters, 17.2/10,000 came to the hospital after delivery for depressive symptoms. Among those with 1 encounter, this rate doubled to 34.9/10,000, and with three or more encounters it increased 7-fold to 126/10,000. The diagnoses for which women came to the hospital prenatally were similar across all populations and the frequency of having a hospital encounter for a mental health condition was similarly low (about 12% of all prenatal visits recorded in our dataset). Of the 20 most common CCS conditions prompting hospital encounters during pregnancy, 16 were shared by all three groups with the remaining four representing mental health disorders in the depression group (*Miscellaneous mental health disorders*, *Mood disorders*, *Schizophrenia and other psychotic disorders*, and *Substance-related disorders*). Although the great majority of prenatal visits were for general medical conditions associated with pregnancy, such as *Hemorrhage during pregnancy*, *Early or threatened labor*, and *Hypertension complicating pregnancy*, visits for mental health conditions were strongly associated with subsequent depression, with rates increasing 18-fold to 653/10,000.

### Conditional probabilities and screening

Because our datasets are not samples but contain the universe of all women seen in hospitals, in these states, over these years, we can directly calculate the conditional probabilities within this population. As presented above, blended data from California and Florida yields a base severe depression rate of 33.9/10,000, which increases significantly to 126/10,000 for patients with 3 or more hospital visits. In the case of a prior mental health hospital encounter, the increase is even more significant, increasing 12-fold for a single mental health encounter during pregnancy (407/10,000, or 4.1%), and more than 65 times if the patient was admitted or visited the ED three or more times during pregnancy for mental health conditions (22.3%). Moreover, additional knowledge of insurance status also impacts the rates of depression. In particular, women with private insurance were almost three times less likely to have a hospital encounter for depression (17.8/10,000) compared to uninsured women or women covered under public insurance (50.3/10,000).

**Fig 4:**
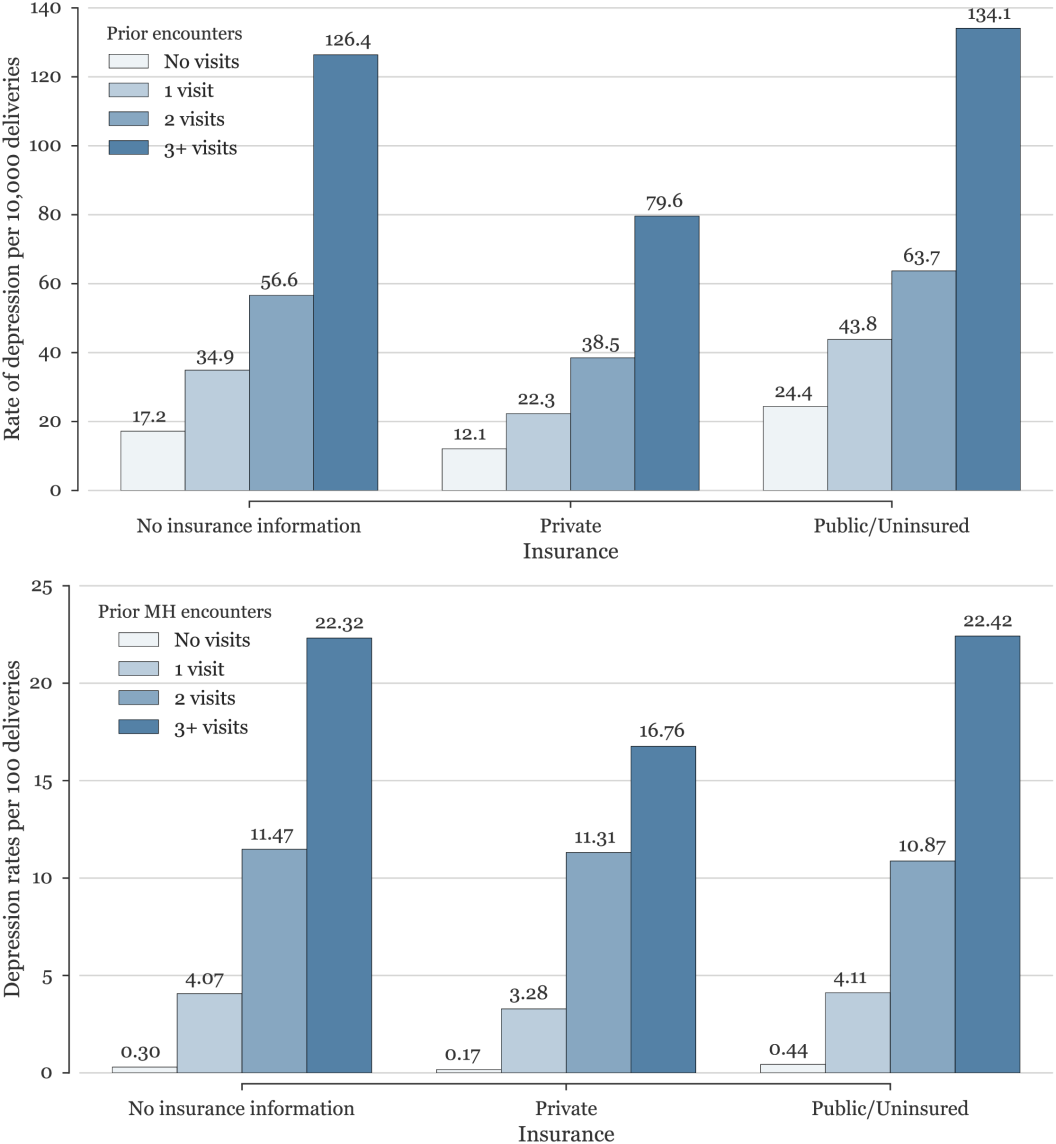
**(Top panel)** Rates of postpartum depression among women with differing insurance status as influenced by the number of prenatal hospital encounters. **(Bottom panel)** Rates of postpartum depression among women of differing insurance status as influenced by number of prenatal hospital encounters *for mental health conditions*. Note scale change: top rates are per 10,000 deliveries, bottom rates are per 100 deliveries.

Combining both sets of prior knowledge demonstrates the range of potential rates depending on prior information when screening different populations. Women with no prior visits and with private insurance have the lowest rate of depression hospital encounters (12.1/10,000), while the knowledge that the woman is uninsured or under public insurance with three or more hospital encounters for mental health during pregnancy increases this rate almost two hundred-fold to 22.4%. Rates of severe depression for all women given various combinations of information are presented graphically in Fig 4 and Table 2.

## Discussion

We defined severe postpartum depression as that marked by symptoms leading to inpatient or outpatient hospital care and then used HCUP data to determine the incidence of severe postpartum depression among 2.6 million women and 3.2 million births. We calculated an overall rate of 36.7/10,000 in 2010 and observed a 34% increase from 27.4/10,000 since 2006. Using synthetic identifiers, we tracked individuals for 40 weeks pre- and post-delivery, observing that hospital encounters for depressive diagnoses were highest in the first month after delivery but continued steadily for 40 weeks thereafter. We also observed that women who had hospital encounters for any reason prior to delivery were much more likely to require hospital care for depression after delivery. Although pre-natal visits for mental health issues most strongly predicted port-delivery depression, regular medical visits were also associated with post-delivery depression in a “dose-dependent” fashion. Finally, uninsured women and those with public insurance were much more likely to appear in hospitals for depression diagnoses than were women with private insurance.

We attempted to focus upon serious postpartum depression by studying only those women who were treated in emergency rooms or admitted to hospitals for depression. Our calculated rates are therefore most comparable to those of Savitz, et al. [8], who observed a 1-year postpartum depression admission rate of 25.6/10,000 in New York state during the 1995 to 2004 decade. Work such as this can also be compared and contrasted with a variety of other studies reporting much higher rates ascertained through screening tools [11, 12], population-based surveillance system [13], or with inclusion of all mental health conditions within different populations [14]. In general, hospital visit rates must underestimate the true incidence and prevalence of depression in a community since many, perhaps most, women are treated outside of hospitals or receive no care at all. In particular, the trends reported here differ from the ones obtained by the survey-based Pregnancy Risk Assessment Monitoring System (PRAMS), which sampled monthly 100–300 new mothers in 27 reporting states [13]. These results are not directly comparable, however, as we report an increase in hospital encounters due to severe postpartum depression but have no information that allows us to make inferences about trends in maternal depression outside of these settings. The increase we report could reflect not only changes in severe postpartum depression requiring hospital attention but also changes in access to care over time in these states. Nevertheless, we believe that rates derived from large hospital datasets can serve as lower bound estimates, trend indicators, and national targets for intervention.

While the frequency of hospital encounters may underestimate the frequency of all depression, their timing of hospital encounters should reflect that of the underlying pathology. We observed a large increase in hospital visits for depression very soon after delivery and then continued presentations for the next 40 weeks. Although roughly half of all visits specifically coded as PPD occurred before the end of the first postpartum month, and 70% by the end of the second, this likely reflects diagnostic labeling conventions. However, visits for all depression were also highest during those periods and then continued steadily at elevated rates for the entire observation period. The early onset of depressive symptoms has been observed elsewhere [15] but, to our knowledge, the persistent elevation above all prenatal mental health visits has not.

The inclusion of emergency room data and the presence of patient identifiers allowed us to extend prior studies in search of postpartum depression antecedents. For this, we aggregated all data over all years and examined pooled rates of associated conditions. Our observation that hospital visits for depression are often preceded by pre-natal visits for other problems (both for mental health and non-mental health conditions) is novel but consistent with findings from a variety of smaller studies associating problems during pregnancy with postpartum depression. For example, Dietz et. al found that 54.2% of women diagnosed with postpartum depression were also identified with depression during pregnancy [16], and Räisänen found that adverse pregnancy events and fear of childbirth were important predisposing factors for depression [17], while Orr and Miller observed an association between depression and preterm birth [18]. The strong association of post-delivery depression and pre-delivery psychiatric symptomatology has also been suggested elsewhere [12].

This study has several limitations. First, any work involving administrative datasets is subject to recording errors and to both geographical and temporal variations in coding practice and to changes in diagnoses awareness by providers and patients. In this regard, the HCUP datasets offer a reliable source of hospital information and the consistency of results across the states we studied as well as an earlier report from New York [8] is reassuring. Second, each dataset is state-based and subject to losses related to interstate migration (i.e. we cannot capture delivering mothers who experience symptoms after leaving the birthing state or symptomatic women who have given birth elsewhere). Third, as discussed above, the rates calculated here can only represent a lower bound for actual community rates since they are limited to hospital encounters. Even so, large scale interventions to detect and treat postpartum depression might reasonably be expected to lower these rates. Fourth, although symptoms requiring emergency room care or hospitalization are usually severe, we cannot distinguish between illness severity and other reasons for visiting hospitals including convenience, lack of alternative care sites, or systemic difficulty in accessing outpatient services. Since visits to hospitals depend on both underlying pathology and the availability or desirability of alternative care sites, differences among states, geographic regions, and ethnic or socioeconomic groups may partly reflect factors other than illness severity. Fifth, we calculated conditional probabilities among the women whose hospital encounters were included in our data, but extrapolation to other regions, time periods, and contexts must be made with caution.

In summary, this work makes the following contributions to screening strategies for postpartum depression: (1) it defines a rate for hospital PPD care at 36.7/10,000 births and suggests that this rate is increasing, (2) it verifies that hospital care for PPD is most frequently sought very soon after delivery in both ED and inpatient settings, but continues steadily for at least 40 more weeks, (3) it observes that women with PPD were frequently treated in ED’s and hospitals for a variety of conditions during their pregnancies, and (4) it quantifies the degree to which insurance status and preexisting mental illness are associated with PPD and underscores the importance of screening for mental health conditions during prenatal ED visits and hospital encounters. Taken together, our findings suggest that postpartum depression screening by primary care physicians, such as by pediatricians during well-child visits, should begin early, should continue for the first year after delivery, and should give added attention to women who have had hospital encounters during their pregnancy.

## Abbreviations

CCS: Clinical Classifications Software
HCUP: Healthcare Cost and Utilization Project
ICD-9-CM: International Classification of Diseases, Ninth Revision, Clinical Modification
PPD: Postpartum depression

## References

[1] Shonkoff JP, Duncan GJ, Yoshikawa H, Guyer B, Magnuson K, Phillips D. Maternal Depression Can Undermine the Development of Young Children: Working Paper No. 8. 2009:1–16.

[2] Siu AL, US Preventive Services Task Force (USPSTF), Bibbins-Domingo K, et al Screening for Depression in Adults: US Preventive Services Task Force Recommendation Statement. *JAMA*. 2016;315(4):380–387. doi: 10.1001/jama.2015.18392 26813211 10.1001/jama.2015.18392

[3] Earls MF, The Committee on Psychosocial Aspects of Child and Family Health. Incorporating Recognition and Management of Perinatal and Postpartum Depression Into Pediatric Practice. *Pediatrics*. 2010;126(5):1032–1039. 20974776 10.1542/peds.2010-2348

[4] Wachino V. Maternal Depression Screening and Treatment: a Critical Role for Medicaid in the Care of Mothers and Children *CMCS Informational Bulletin*, *Department of Health and Human Services*, *Centers for Medicare and Medicaid Services*, Baltimore; 2016:1–6.

[5] Stewart DE, Vigod S. Postpartum Depression. *N Engl J Med* 2016; 375:2177–2186 doi: 10.1056/NEJMcp1607649 27959754 10.1056/NEJMcp1607649

[6] Myers ER, Aubuchon-Endsley N, Bastian LA, Gierisch JM, Kemper AR, Swamy GK, et al. Efficacy and Safety of Screening for Postpartum Depression. Comparative Effectiveness Review 106. (Prepared by the Duke Evidence-based Practice Center under Contract No. 290-2007-10066-I.) AHRQ Publication No. 13-EHC064-EF. Rockville, MD: Agency for Healthcare Research and Quality; April 2013. www.effectivehealthcare.ahrq.gov/reports/final.cfm.

[7] HCUP State Inpatient (SID), Emergency Department (SEDD), and Ambulatory Services (SASD) Databases. Healthcare Cost and Utilization Project (HCUP). 2005–2012. Agency for Healthcare Research and Quality, Rockville, MD https://www.hcup-us.ahrq.gov/databases.jsp. Accessed on: June 14, 2016.

[8] Savitz DA, Stein CR, Ye F, Kellerman L, Silverman M. The Epidemiology of Hospitalized Postpartum Depression in New York State, 1995–2004. *Annals of Epidemiology*. 2011;21(6):399–406. doi: 10.1016/j.annepidem.2011.03.003 21549277 10.1016/j.annepidem.2011.03.003 PMC3090997

[9] Perkel JM. Programming: Pick up Python. *Nature*. 2015;518(7537):125–126. doi: 10.1038/518125a 25653001 10.1038/518125a

[10] Perez F, Granger BE. IPython: A System for Interactive Scientific Computing. *Comput Sci Eng*. 2007;9(3):21–29. doi: 10.1109/MCSE.2007.53

[11] Josefsson A, Berg G, Nordin C, Sydsjö G. Prevalence of depressive symptoms in late pregnancy and postpartum. *Acta Obstetricia et Gynecologica Scandinavica*. 2001;80(3):251–255. doi: 10.1034/j.1600-0412.2001.080003251.x 11207491 10.1034/j.1600-0412.2001.080003251.x

[12] O'Hara MW, Zekoski EM, Philipps LH, Wright EJ. Controlled prospective study of postpartum mood disorders: comparison of childbearing and nonchildbearing women. *J Abnorm Psychol*. 1990;99(1):3–15. 2307763 10.1037//0021-843x.99.1.3

[13] Ko JY, Rockhill KM, Tong VT, Morrow B, Farr SL. Trends in Postpartum Depressive Symptoms—27 States, 2004, 2008, and 2012. MMWR. Morbidity and Mortality Weekly Reports, 2017. Retrieved from https://www.cdc.gov/mmwr/volumes/66/wr/mm6606a1.htm. Accessed on Sept 15, 2017. 10.15585/mmwr.mm6606a1 PMC5657855 28207685

[14] Xu F, Sullivan EA, Li Z, Burns L, Austin M-P, Slade T. The increased trend in mothers' hospital admissions for psychiatric disorders in the first year after birth between 2001 and 2010 in New South Wales, Australia. *BMC Womens Health*. 2014;14(1):119 doi: 10.1186/1472-6874-14-119 25263987 10.1186/1472-6874-14-119 PMC4261248

[15] Xu F, Sullivan E, Binns C, Homer CSE. Mental disorders in new parents before and after birth: a population-based cohort study. *British Journal of Psychiatry Open*. 2016;2(3):233–243. doi: 10.1192/bjpo.bp.116.002790 27703780 10.1192/bjpo.bp.116.002790 PMC4995171

[16] Dietz PM, Williams SB, Callaghan WM, Bachman DJ, Whitlock EP, Hornbrook MC. Clinically Identified Maternal Depression Before, During, and After Pregnancies Ending in Live Births. *Am J Psychiatry*. 2007; 164(10): 1515–1520. doi: 10.1176/appi.ajp.2007.06111893 17898342 10.1176/appi.ajp.2007.06111893

[17] Räisänen S, Lehto SM, Nielsen HS, Gissler M, Kramer MR, Heinonen S. Fear of childbirth predicts postpartum depression: a population-based analysis of 511 422 singleton births in Finland. *BMJ Open*. 2013;3(11):e004047 doi: 10.1136/bmjopen-2013-004047 24293208 10.1136/bmjopen-2013-004047 PMC3845069

[18] Orr ST, Miller CA. Maternal depressive symptoms and the risk of poor pregnancy outcome. Review of the literature and preliminary findings. *Epidemiol Rev*. 1995;17(1):165–171. 8521934 10.1093/oxfordjournals.epirev.a036172

